# Impact of Forest Management on the Temporal Dynamics of Herbaceous Plant Diversity in the Carpathian Beech Forests over 40 Years

**DOI:** 10.3390/biology10050406

**Published:** 2021-05-05

**Authors:** Anna Bugno-Pogoda, Roma Durak, Tomasz Durak

**Affiliations:** 1 Laboratory of Plant Physiology and Ecology, University of Rzeszów, Rejtana 16C, PL-35-959 Rzeszów, Poland; anna.bugno.pogoda@gmail.com; 2 Faculty of Agriculture and Forest Management, The Jan Grodek State University in Sanok, Mickiewicza 21, PL-38-500 Sanok, Poland; 3 Department of Experimental Biology and Chemistry, University of Rzeszów, Pigonia 1, PL-35-310 Rzeszów, Poland; rdurak@univ.rzeszow.pl

**Keywords:** alfa diversity, biotic homogenization, long-term interactions, change of the forest management system, forest conservation status, forest developmental stages, shelterwood silvicultural systems

## Abstract

**Simple Summary:**

Vegetation composition and plant diversity of mixed deciduous forests in Europe is strongly linked with the dynamics of the forest stand and/or the regimes of forest management. In this work, we showed the influence of temporal interactions among the changes in the management system—the dominant development stage—the intensity of forest treatments, and herbaceous plant diversity. We argued that different arrangements of these interactions will result in different patterns of change in herbaceous plant diversity. We emphasized the need for careful interpretation of the levels of diversity (α, β) to evaluate the conservation status of forests.

**Abstract:**

In recent years, there has been a growing awareness of the complex dependence of herbaceous plant diversity on forest structure and management. However, among the studies presented so far, those in which the chronosequence (approach based on the assumption of space-for-time substitution) was used, dominate. On the other hand, it is rare to find results based on long-term research on permanent or semi-permanent sampling plots. The aim of this study was to recognize the changes in the vegetation composition and dynamics of various indices of herbaceous plant diversity over 40 years of forest development, and their dependence on forest structure and management. Here we analyzed the temporal dynamics of herbaceous plant diversity in Carpathian fertile beech forests, based on datasets recorded on semi-permanent plots in three censuses (the 1970s, 2000s and 2010s). We checked the temporal changes in alpha, beta, and gamma diversity. Analyses of the plant diversity were performed on the background of changes in forest structure and management systems. We found that the within-plot (alfa diversity) and between-plot (beta diversity) herbaceous plant diversity metrics showed inconsistent patterns along with changes in the forest structure, management systems, and intensity of forest management, during the last 40 years. Temporal changes in the gamma diversity followed the changes in alpha diversity. Although the beta diversity after 40 years is greater than in the past, we argue that the conservation status of habitats typical for well-preserved fertile mountain beech forests has deteriorated due to a decline in the sharing of the diagnostic species of these forests. We showed the importance of the different temporal interactions between the forest structure and management for herbaceous plant diversity. We argue that, in view of the complexity of these processes, it would be a mistake to reject or prioritize alpha or beta diversity measurements to determine the real course of long-term changes in herbaceous plant diversity and to properly assess the state of the forest biodiversity, their conservation status, or conservation action plans. In addition, we need far more data from long-term observations to fully understand the possible relationship patterns between the factors controlling the forest structure and plant diversity.

## 1. Introduction

In temperate forests, the herbaceous layer constitutes most of the forest plant diversity and has significant influence on key ecosystem processes, such as nutrient cycling, tree regeneration, and competitive interactions [1]. However, since the second half of the twentieth century, the species composition of European forest herbaceous plant communities has experienced rapid changes, which, very often, have been identified as a threat to the functioning of plant communities and ecosystems and have led to the loss of biodiversity [2,3,4]. Changes in species composition were caused by the processes changing the environmental conditions, which resulted in the disappearance or spread of species. These changes in the environmental conditions were most often driven by global changes (especially climate changes and air pollutions) or forest management and noncommercial forest human use, such as collecting firewood and raking litter [5,6]. They often led to the spread of habitat generalists or invasive species and the disappearance of habitat specialists. The latter were associated especially with the loss of nitrogen-poor habitats and an increase in temperature. As a result, changes in forest herbaceous plant communities were frequently recognized as directional processes, resulting in a simplification of their species structure and a loss of diversity on different levels of spatial organization [7,8].

Recently, forest ecologists have revealed an increasingly complex picture of the impact of forest structure and forest management on the species composition and diversity of communities [9,10,11,12]. It has been shown that the composition of forest communities or diversity were not only dependent on the stands’ differentiation in terms of their structure specific for different developmental stages/phases but, also, from the forester-shaped spatial distribution of the stands’ developmental stages, as well as the size of the patches occupied by the forest developmental stages/phases. For example, Schall et al. [13] indicated that, on a regional scale, coarse-grained management (shelterwood system) by creating a mosaic of stands in different age classes, can harbor more biodiversity than fine-grained selection systems (resulting in higher within-stand heterogeneity, but low between-stand). Hilmers et al. [10] highlighted the high dependency between the forest developmental stages and biodiversity and underlined that the cyclical impact of forest dynamics (internal drivers) can be misinterpreted as directional impact of the external drivers (e.g., climate change).

In recent decades, in addition to forest management as such, the vertical and horizontal structures of forests are significantly influenced by shifts between the forest management system, implemented to replace intensive forest management strategies with close-to-natural methods [14,15,16,17]. Thus, shifts between the forest management system were used to convert low forests (known as coppice forests) into high forests (stands consisting of large, tall, mature trees with a closed canopy). In the case of such conversion of a forest stand, large changes in herbaceous plant diversity could be expected. Indeed, many authors have reported a decrease in the structural and functional plant diversities, as well as in the conservation values of forests [18,19,20]. However, in the case of changes in the management system in high forests (e.g., changes from clear-cutting to shelterwood or selective-cutting systems), the changes in the forest herbaceous layer are less-recognized. Additionally, less obvious changes can be expected in the forest herbaceous layer than in the case of a converted low forest. For example, Durak and Holeksa [21] found biotic homogenization in forest herbaceous communities in resource-rich habitats and biotic differentiation in resource-poor habitats in aging Carpathian beech forests, where regular shelterwood has been changed to an irregular shelterwood system. These observations are important in the context of the development of age structure of forests throughout Europe. Although the majority of these forests are currently of intermediate age, the proportion/area of late forest developmental stages (e.g., terminal stage) is clearly growing [22]. It follows that the unravelling of the influence of the stand structure on changes in the composition and diversity of the forest herbaceous layer should be considered within the context, of course, of changes in the forest management and stand structure. It should be expected that not only the change in the management method be important; we argue that the impact of change in the management system will depend on the time point in the forest development when the management system was changed, as well as from the developmental stage itself.

Long-term research seems to be the most reliable way of unravelling vegetation responses to temporal changes [23,24]. However, so far, the knowledge gained in the relationship between changes in the forest structure and management (on the one hand) and herbaceous vegetation composition and plant diversity (on the other hand) was mostly based on vegetation records from two time points (e.g., [19,25,26,27]). Here, we analysed the dynamics of the Carpathian fertile beech forest herbaceous plant diversity (managed semi-natural forests) based on datasets recorded on semipermanent plots in three subsequent censuses (the 1970s, 2000s, and 2010s).

So far, due to the simplicity of the measurements, the effects of forest management on species diversity were studied mostly with the focus on alfa diversity expressed by species richness [28,29,30]. However, there are three diversity components that depend on the spatial scale: the aforementioned local alpha diversity (measured within locations, in this work—within stands), as well as beta diversity (measured between locations, in this work—between stands) and regional gamma diversity. Beta diversity is known as a fundamental component of biodiversity—decisive for the process of biotic homogenization (decrease of beta diversity, resulting in an increase in species similarity across space over time) [31,32]. The term gamma diversity is defined as the regional species pool. It is assumed that these three diversity components are related to each other and that beta diversity provides a link between the local alpha diversity and regional gamma diversity [33,34,35]. It transpires, however, that the dependencies between diversity components are not always obvious. For example, Schall et al. [13] showed that the local alfa diversity may hide patterns of remaining diversity components (beta and gamma diversity). Moreover, alfa diversity adopted as an indicator for the conservation status of the forests can lead to the wrong conclusion [11]. Hence, in order to better understand the patterns of herbaceous vegetation in beech forests, we examined all three diversity components. Moreover, to get a sound and relevant measure of alfa diversity as a measure of the conservation status of the forests, in addition to classical alfa indices (Shannon and evenness diversity indices), we expressed alfa diversity as a species richness of ecological groups of species defined as species specialists with high and low habitat requirements. Additionally, to recognize the conservation status of investigated forests, we took into account conservation-relevant species (diagnostic species for beech forests), as well as plant species whose frequencies of occurrence or abundance increased significantly (hereafter, winner species) or decreased (hereafter, loser species) throughout the research period.

Analyses of the herbaceous plant diversity were performed on the background of changes in the stand structure (resulting from the forest stands’ development) and management system (from the 1990s, regular shelterwood was replaced by close-to-nature irregular shelterwood silvicultural systems). Therefore, we expected to find a more complex answer to the course of changes that have occurred in herbaceous plant diversity during the past 40 years and their drivers.

We expect that the increased or decreased differentiation of the spatial structure of the forest (forest habitat heterogeneity) will result in a decrease or increase in the resource availability (trade-off between the area available for individual species and habitat heterogeneity on the spatial scale) according to the “area heterogeneity trade-off hypothesis” [36]. Consequently, this may result in a reduction or increment in the size of local populations and an increase or decrease in the likelihood of their stochastic extinction, which will be reflected in the different diversity patterns between vegetation censuses.

The aim of this study was to recognize: (1) temporal dynamics of the herbaceous plant diversity (alpha, beta, and gamma diversity) over 40 years of forest development and management; we expected inconsistencies in the patterns of diversity within and between the vegetation censuses; (2) the relationships between forest structure and management and herbaceous plant diversity on different spatial scales; (3) the contribution of ecological groups of the species to the reaction of herbaceous vegetation to changes in the forest structure and forest management; and the (4) impact of the changes in forest structure and management on the species composition, taking into account winner and loser species, as well as species having important conservation statuses in fertile mountain beech forests (FMBF).

## 2. Materials and Methods

### 2.1. Study Area

**Figure 1:**
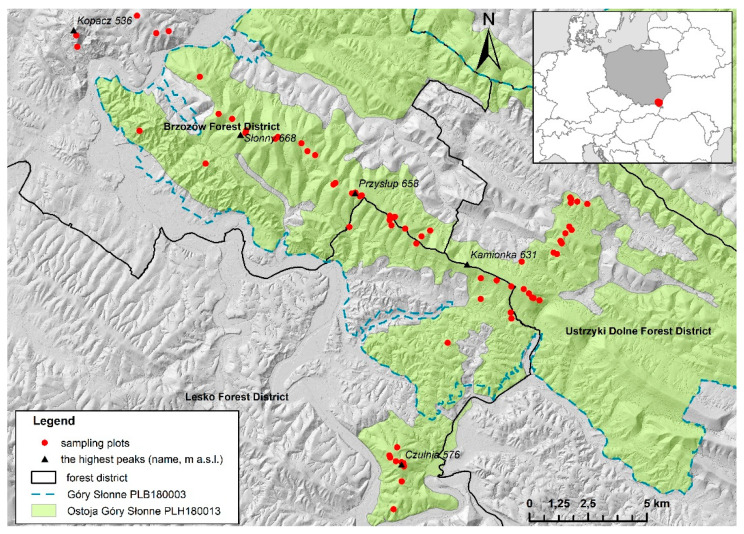
Location of the study site and distribution of semipermanent sampling plots within the study area.

The study area is located in the Sanocko-Turczańskie Mountains in the Polish Eastern Carpathians (49°33 6.900″ N; 22°20 42.225″ E; Figure 1). This part of the Polish Carpathian Mountains is dominated by brown soils formed from Carpathian flysch [37,38]. The average annual temperature is 7.7 °C, and the annual rainfall is 820.8 mm (data from the station Lesko, 420 m a.s.l., for the period 1966–2018 [39]). The forests are dominated by FMBF (according to the phytosociological classification—*Dentario glandulosae Fagetum* Klika 1927 em. Mat. 1964). The dominant species in these forests is European beech (*Fagus sylvatica*). Moreover, silver fir (*Abies alba*) and sycamore maple (*Acer pseudoplatanus*) may appear in small admixtures. This area is part of the Natura 2000 network (“Ostoja Góry Słonne” PLH180013 and “Góry Słonne” PLB180003).

Forests in this region are managed by the Brzozów, Lesko, and Ustrzyki Dolne Forest District. Between the 1950s and 1990s, the forests were regenerated using the regular shelterwood system, the most popular cutting method in the mountainous area of the Polish Carpathians [40]. The regular shelterwood system created even-aged stands with low variation in the tree sizes. At the end of the 1990s, the management system was changed to an irregular shelterwood system, which more efficiently imitates the natural processes occurring in forest ecosystems, creating a closer to nature forest structure (it produces irregular stand structures, even on small spatial scales). This management system, compared to the regular shelterwood system is characterized by an extended rotation age (110–130 years) and a longer regeneration period (from 30 to 50 years). For a more detailed comparison of the two forest management systems, please refer to Table 1.

### 2.2. Data Collection

In our study, three sets of vegetation records were compared. Vegetation records were made according to the Braun-Blanquet method [41] as so-called phytosociological relevés, during three vegetation censuses (1972–1973, 2005–2007, and 2017–2018) on an irregular network of 67 sampling plots (Figure 1). During the second vegetation census, these plots were reestablished, marked by geographic coordinates, and resurveyed [21]. Geographic coordinates of the sampling plots made it possible to locate them extremely accurately during the third vegetation census. After the re-localization of sampling plots, their location was additionally verified using descriptions from the 1970s. We found compliance in the case of all plots. To make the three datasets comparable, during both resurveys, the vegetation records were taken from 67 sampling plots with the same area (usually 200 or 400 m^2^) during the growing season, as in the 1970s. Vegetation records contain data on: forest layer coverage, species composition of forest layers, and abundance of plant species in individual forest layers (estimated using the cover–abundance scale). In addition, they contained data on the prevalent height and DBH of trees in the sampling plot. Forest layer coverage and height of trees were measured according to the Braun-Blanquet [41] methodology. Under this approach, the coverage of the tree and shrub layers was estimated on the basis of their vertical projections as a percentage of the sampling plot. Tree height was defined as the most common tree height in the tree layer and was determined based on one measurement per sampling plot. The DBH of a tree (measured at a height of 1.3-m aboveground level) was defined as the most common DBH of a tree in the sampling plot and determined similarly to the height of the tree. Moreover, datasets from the vegetation records were expanded to include the age of stands and the forest management intensity included in the Forest Management Plans as of 1976–1977, 2007–2009, and 2017–2019 (by forest sub-compartments on which individual sampling plots were located). The management plans used were prepared for Forest Districts Brzozów, Lesko, and Ustrzyki Dolne (available in the Regional Directorate of the State Forests in Krosno).

### 2.3. Data Analysis

In order to ascertain the relationship between forest structure and management and the diversity dynamics of the herbaceous layer, we considered forest structure characteristics, stand age, and intensity of forest management, as well as changes in the forest management system. Forest structure characteristics were measured on the sampling plot level. Tree and shrub (including tree and shrub saplings) layer cover (%), tree height and DBH, and number of species in tree and shrub layers were recorded. Due to a lack of DBH data in records from the 1970s, they were supplemented by data from the oldest available forest inventories (Forest Management Plans from the 1970s). Prior to that, we checked whether the forest inventory data corresponded to the data measured on the sampling plots. For this purpose, we used data from the 2000s. We calculated the average DBH for the data from the sampling plots and corresponding inventory data (average DBH, 49.0 cm and 48.4 cm, respectively). Additionally, we correlated them (r_s_ = 0.32, *p* ≤ 0.01). Based on the results, we found that the inventory data sufficiently corresponded to the data from the sampling plots. Moreover, based on Forest Management Plans from the 1970s, 2000s, and 2010s, the age of the stands (mean age for the dominant tree species), as well as the forest management intensity levels, were considered. To reveal changes in the intensity of forest management, the management intensity levels were arranged from the lowest to the highest and ranked on a five-point scale. To emphasize the importance of low, as well as a very high intensity of forest management, the points on this scale were not equidistant: no interference—1, thinning—4, irregular shelterwood treatments lasting 10 years—6, irregular shelterwood treatments lasting 20 years—7, and regular shelterwood treatments—9. All data from the Forest Management Plans were compiled for forest sub-compartments (the basic territorial unit of the State Forests, defined for the needs of forest management) in which the sampling plots were located.

We considered changes in the alpha, beta, and gamma herbaceous plant diversities. For this purpose, we computed the Shannon and evenness diversity indices (α_Sha_ and evenness), Sorensen dissimilarity index (β_So__r_, total beta diversity) partitioned according to Baselga [42] on the Simpson dissimilarity index (β_Sim_, species turnover component), and nestedness (β_nes_, species extinction or colonization component). Gamma diversity (γ) was defined as the total species pool noted in a given census.

To detect the biotic homogenization or differentiation of the herbaceous vegetation, a method based on the average inter-plot dissimilarities was adopted [25,43,44]. Changes were computed as the difference in the pairwise species dissimilarities between the distinct sampling times [45]. To accomplish this, dissimilarity indices (β_Sor_, β_Sim_, and β_nes_) were calculated, for all possible pairs of plots from the 1970s, 2000s, and 2010s. Then, the average dissimilarity indices were calculated for each plot in each of the three vegetation censuses. Differences between the vegetation censuses were evaluated using a several sample repeated-measure ANOVA test with posteriori Tukey’s test. We assumed that a decrease or increase in the mean values of the β_Sor_ index would indicate the homogenization or differentiation of vegetation over time. Concurrently, both components of β_Sor_ that quantify the species turnover (species replacement) and nestedness (species richness) can change opposite to each other.

This method, based on the average inter-plot dissimilarities, was also adopted to detect changes in the forest habitat heterogeneity between plots. To reveal the habitat conditions prevailing on sampling plots, we used Ellenberg indicator values (EIVs) for light (L), temperature (T), soil moisture (F), soil reaction (R), and soil nitrogen (N) [46]. Average indicator values were calculated using plant species qualitative (presence/absence) data for sampling plots from each vegetation census. In order to quantify the changes in habitat heterogeneity between the 1970s, the 2000s and 2010s, the abundance-based Morisita-Horn (M-H) dissimilarity index was used [47]. In the case of finding changes in the habitat heterogeneity in one of the study periods, we checked their relationship with changes in the herbaceous plant diversity within-plot (∆α_Sha_) and between-plots (∆β_Sor_). For this purpose, the correlation between ∆α_Sha_ and ∆β_Sor_ indices and the changes in the M-H coefficients (expressing habitat heterogeneity) in the relevant study period were tested.

At the community level, the herbaceous species frequency of occurrence was compared between the vegetation censuses. Herbaceous species richness, richness of ecological groups of species defined based on high or low requirements for habitat conditions (estimated on the basis of Ellenberg indicator values), and total abundance (sum of abundances of species estimated by the Braun-Blanquet cover-abundance scale transformed to mid-point percentage values) were considered at plot level for each vegetation census. Among the distinguished ecological groups of species were groups of species with high (EIVs ≥ 7, L_H_, F_H_, R_H_, and N_H_) and low indicator values (EIVs ≤ 3, L_L_, R_L_, and N_L_). Due to the small number of the species meeting the criteria, we did not consider the F_L_ group, and the T_H_ and T_L_ were expanded to include species with indicator values ≥ 6 and ≤ 4, respectively. At the species level, by comparing the frequency of the occurrence and species abundance between vegetation censuses, we identified the winner and loser plant species groups. To these groups, we included only plant species for which (1) the sum of changes in the frequency of occurrence in the first and second study periods increased or decreased by at least 10%, or (2) the abundance between the three vegetation censuses revealed a significant increase or decrease. Moreover, to detect the changes in species important from a conservation status FMBF point of view, the diagnostic species for beech forests in the phytosociological system of plant communities (diagnostic species of alliance *Fagion sylvaticae* and association *Dentario glandulosae Fagetum*, [48]) that underlie the Habitat Directive for the Natura 2000 program sites in the EU were taken into account. In this case, we studied the temporal trend in the occurrence of these species.

Differences between the scores obtained based on three vegetation censuses were tested using several sample repeated measures ANOVA or Friedman tests with posteriori Tukey’s or Wilcoxon’s tests with Bonferroni correction, respectively. ANOVA test was used when the data met the assumption of normality. Otherwise, the Friedman test was used. To reveal dependencies between changes in the stand structure, as well as the intensity of forest management and diversity indices and groups of species, the Spearman’s rank correlation test was applied. Unless stated otherwise, statistical significance was estimated for *p* ≤ 0.05.

To avoid the overestimation of common species of high abundance, and to improve the normality of distribution, before statistical analyses, all data were square root-transformed. In order to avoid errors resulting from the differences in herb layer compositions due to shifts in the spring season (in response to global change), early spring herbaceous species were excluded from the analyses. In order to avoid errors resulting from the incorrect identification of similar species, ferns of the genus *Dryopteris* were combined into one group. The same applied to *Senecio fuchsii* and *S. nemorensis*.

All statistical analyses were calculated using the PAST software package 4.0 (Hammer et al. [49]).

## 3. Results

### 3.1. Dynamics of Change in the Forest Structure and Intensity of Forest Management

The stand age increased from the first to the third census. In both study periods, the tree layer cover decreased and shrub layer cover increased. However, only in the second study period were these changes statistically significant. The highest average tree height was noted during the second census. However, this was not significantly different from the tree height noted during the first census, and both were statistically higher than the average tree height in the third census. The average DBH statistically increased in the first study period and decreased in the second. Species richness of the tree layer showed an increase, and species richness of the shrub layer decreased from the first to third censuses (Table 2).

**Figure 2:**
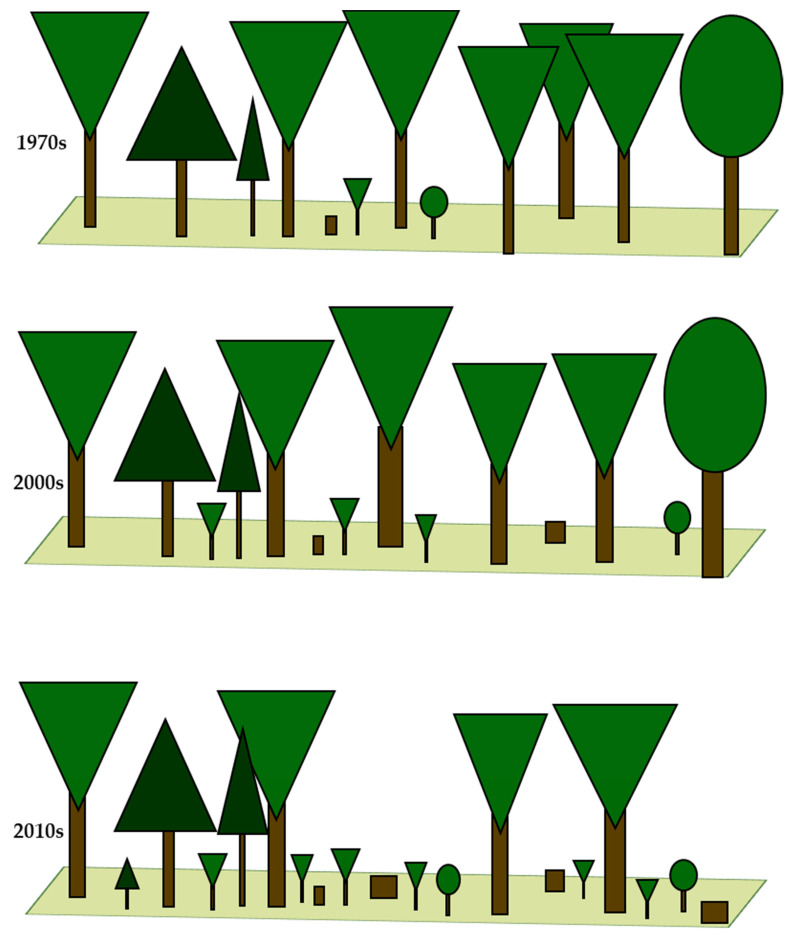
Schematic representation of the vertical and horizontal structures of fertile mountain beech forests in the 1970s, 2000s, and 2010s.

**Figure 3:**
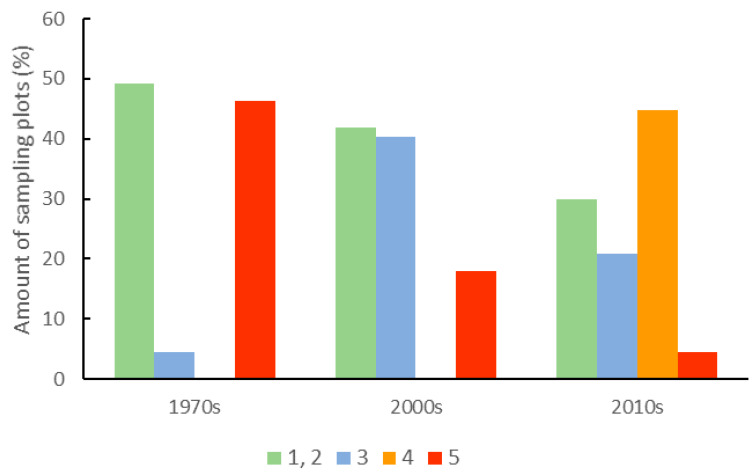
Forest management treatments on sampling plots in the three subsequent vegetation censuses. 1—no interference, 2—thinning, 3—irregular shelterwood treatments lasting 10 years, 4—irregular shelterwood treatments lasting 20 years, and 5—regular shelterwood treatments.

Between the 1970s, and 2000s, we observed significant changes in the intensity of forest management. In the 1970s, over 45% of sampling plots were located in stands without or under small forest management pressure. However, at the same time, a similar percentage of sampling plots were located in stands subject to severe forest management treatments. In the 2000s, the severity of forest management decreased due to the replacement of regular shelterwood treatments to irregular shelterwood treatments. In the 2010s, most stands achieved the rotation age. As a result, there was a substantial decrease in the number of sampling plots with stands without or under small forest management pressure. At the same time, the number of sampling plots with stands subject to long-term, irregular shelterwood treatments with clearly visible stand renewal processes increased (Table 2 and Figure 2 and Figure 3).

### 3.2. Dynamics of Change in the Herbaceous Plant Diversity Metrics

The highest gamma diversity, defined as the total species pool noted during each of the censuses, was recorded in the 2000s (131 species). The total pool of species in the 1970s and 2010 was similar and amounted to 117 and 118 species, respectively.

**Figure 4:**
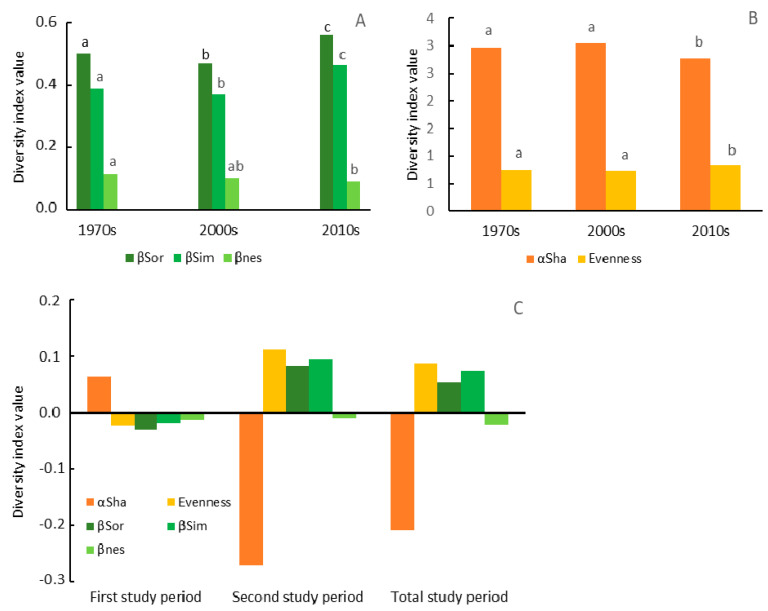
Mean values of the alfa and beta diversity indices in the three subsequent vegetation censuses. (**A**) A pairwise β_Sor_ dissimilarity index partitioned into the β_Sim_ (replacement) and β_nes_ (nestedness) components. (**B**) Alfa diversity indices. (**C**) Changes in the alfa and beta diversity indices during the first, second, and total study periods.

Shannon diversity index was highest in the 2000s and lowest in the 2010s. The evenness index was lowest in the 2000s and highest in the 2010s (Figure 4). Several sample repeated measures tests confirmed a decrease in the Shannon diversity (ANOVA test: F = 13.4, *p* ≤ 0.001) and in the variation in species abundance on the sampling plots in the 2010s (increase in the evenness index, Friedman test: chi^2^ = 54.6, *p* ≤ 0.001).

Beta diversity expressed by the Sorensen and Simpson dissimilarity indices was highest in the 2010s and lowest in the 2000s. Statistical tests confirmed decreased beta diversity indices at the end of the first study period and an increase at the end of the study period (Sorensen: F = 47.1, *p* ≤ 0.001; Simpson: F = 50.3, *p* ≤ 0.001). Beta diversity expressed by the nestedness index, systematically decreased from the 1970s. In the 2010s, it was statistically lower than in the 1970s (F = 3.7, *p* ≤ 0.05) (Figure 4).

Changes in the beta diversity indices indicated homogenization in species composition during the first study period, with a significant decrease in species replacement between sampling plots. During the second period, there was a clear differentiation in species composition, with a significant increase in species replacement between sampling plots. Moreover, the comparison of beta diversity between the 1970s and the 2010s showed the differentiation process with a significant increase in species replacement and a simultaneous decrease in the nestedness beta diversity component (Figure 4).

The forest habitat heterogeneity between plots increased during the 40 years of the study (the M-H dissimilarity index based on data from three subsequent censuses were: 0.0014, 0.0022, and 0.0033). Several sample repeated measures ANOVA tests showed significant differences between these indices (F = 16.9, *p* ≤ 0.001), and the posteriori Tukey’s test confirmed a significantly higher forest habitat heterogeneity in the 2010s than in the 1970s and 2000s. These results indicated that the habitat heterogeneity increased during the second study period. We found a strong correlation between ∆α_Sha_ and ∆β_Sor_ indices and the changes in the habitat heterogeneity (negative, r_s_ = −0.48, *p* ≤ 0.001; positive, r_s_ = 0.47, *p* ≤ 0.001, respectively).

**Figure 5:**
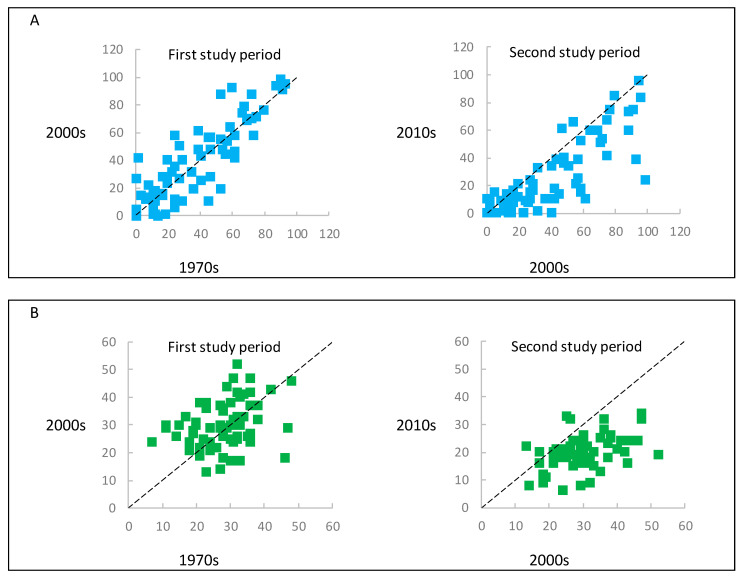
Frequency of species occurrence (**A**), and species richness (**B**) on the sampling plots during the vegetation censuses. For the sake of clarity, diagram A was plotted from species with a frequency of occurrence of at least 10% over any vegetation census. The dashed lines presents a hypothetical situation where the frequency of species occurrence or species richness in the sampling plots were equal between the vegetation censuses.

At the community level, the mean frequency of species occurrence was highest in the 2000s, intermediate in the 1970s, and lowest in the 2010s. Based on the statistical results, the frequency of species occurrence from the 2010s was significantly lower than in earlier censuses. At the plot level, the mean species richness was highest in the 2000s, intermediate in the 1970s, and lowest in the 2010s. The total species abundance was lowest in the 1970s and highest in the 2010s. The statistical results indicated a decrease of species richness and an increase of total species abundance in the 2010s (Table 3 and Figure 5). In the case of the ecological groups of species defined based on the Ellenberg indicator values, we found the highest species richness of groups: L_L_, T_L_, F_H_, R_H_, and N_H_ in the 2000s and lowest in the 2010s. Vegetation censuses differed significantly in the richness of shade-tolerant and moisture-demanding species. The richness of the species, which preferred soil with higher pH and rich in nutrients, as well as cooler habitats, significantly decreased in the 2010s (Table 3).

From the pool of 20 winner species, 16 and 19 species noted changes, respectively, during the first and second study periods. Among them, the highest increase was noted by disturbance-related species with higher light requirements: *Rubus hirtus* and ferns from genus *Dryopteris*. At the same time, from the pool of 45 loser species, a similar number of species noted changes (41 and 42 species), respectively, during the first and second study periods. Among them, the highest decrease was noted for typical, shade-tolerant FMBF species: *Actaea spicata*, *Athyrium filix-femina*, *Daphne mezereum*, *Mercurialis perennis*, *Oxalis acetosella*, and *Polygonatum multiflorum*. Moreover, a large group of diagnostic species of FMBF was found among the loser species: *Dentaria glandulosa*, *D. bulbifera*, *Symphytum cordatum*, *S. tuberosum*, *Euphorbia amygdaloides*, and *Glechoma hirsuta*. Additionally, we found a group of species that decreased in frequency of occurrence and increased in abundance between 1970s and 2010s, e.g., *Galeobdolon luteum* and *Fagus sylvatica* (Table S1).

Between the 1970s and 2000s, only a slight increase in the occurrences of species important from a conservation status of the FMBF viewpoint was found (from 307 to 322 occurrences). The change of the management system, as well as the intensification of forest management, along with the increase in the age of the forest, resulted in a decrease in the occurrence of diagnostic species of FMBF between the 2000s and 2010s (from 322 to 253 occurrences).

### 3.3. Impact of Changes in the Forest Structure, as Well as Intensity of Forest Management on the Herbaceous Plant Diversity Metrics

We did not find dependencies between the changes in the forest characteristics and changes in the herbaceous plant diversity metrics during the first study period.

During the second study period, the herbaceous plant alpha diversity metrics showed a decrease, with an increase in shrub layer cover. The total herbaceous plant beta diversity (β_Sor_) increased with a decrease in the height and number of tree species. Components of the total beta diversity (β_Sim_ and β_nes_) revealed additional, inverse dependency from the cover of the tree layer (Table 2 and Table 4). The changes in the forest structure during the second study period were much greater than in the first period (Table 4). A decrease in the tree layer cover and tree height and increase in the shrub layer cover indicate the intensive felling of old trees and replacement with a new tree generation during the second study period. Therefore, the described-above relationship between the changes in the forest structure and herbaceous plant diversity metrics was attributed to the higher intensity of management treatment in the 2010s.

## 4. Discussion

Based on the stand age and the characteristics of the forest structure, we can define the forest developmental stages that dominated during the three subsequent vegetation censuses. The stand age exceeding 85 years on average, DBH over 30 cm, and the lack of differences between most features of the forest structure from the 1970s and the 2000s indicate the dominance of the optimum forest developmental stage during the first and second vegetation census. [12]. However, the greater DBH of trees, as well as the lower species richness of stands with the simultaneously growing species richness of the shrub layer point to the fact that the stands from the 2000s were dominated by the late optimum stage. The forests from the 2010s were very different compared to those in the 1970s and 2000s. Decline in tree size (height and DBH), tree layer cover, and species richness, with the simultaneous increase in the coverage of the intermediate forest layer (consisting of tree saplings and shrubs), led to the assumption that in the 2010s the terminal stage was dominant [12].

We found that the within-plot and between-plot herbaceous plant diversity metrics showed inconsistent patterns alongside changes in the beech forest structure, management system, and intensity of the forest management during the last 40 years. Our results disclose that change of the dominant developmental stage of the forest from the optimum to late-optimum stage, combined with a change in the management system to a less-invasive, closer-to-nature at the beginning causes an increase in within-plot and a decrease in between-plot herbaceous plant diversity. However, in the following years (2010s, development of terminal stage), when the intensity of forest management combined with forest renewal processes increased, the within-plot herbaceous plant diversity strongly decreased, and the between-plot increased, except for beta nestedness, which decreased. We argued that this increase in beta diversity can be misleading when assessing the status of the forest diversity or planning conservation actions because of a decrease in the alpha diversity metrics, as well as including diagnostic species of beech forests. This loss of species is in accordance with the “area heterogeneity trade-off hypothesis” [36] and results in a slight decline in the diversity on the regional level (gamma diversity) in the 2010s.

### 4.1. Dynamics of Change in the Herbaceous Plant Diversity Metrics

The alpha diversity (α_Sha_, within-plot diversity) only slightly increased during the first study period and substantially decreased in the second period. At the same time, changes in the beta diversity (β_Sor_, as well as β_Sim_, between-plot diversity) between subsequent vegetation censuses were statistically significant, showing a clear nonlinear, U-shaped pattern of changes. Inconsistency between the revealed patterns of herbaceous plant diversity indicates that alpha and beta diversity react inversely to changes of forest management treatments and/or the severity of the forest management system. Moreover, beta diversity is more sensitive to these changes. Opposite patterns of alpha and beta diversity changes can be explained by the inherent relationships between most of the traditional alpha and beta diversity indices [50]. However, we did not find the same clear U-shaped trend in alpha diversity, as was in the case of the beta diversity. This means that alpha diversity reacts to a lesser extent than beta diversity to a decrease in the intensity of forest management treatments. Nevertheless, both alpha and beta diversity react strongly to increases in forest management treatments (decrease in alpha and increase in beta diversity metrics). There are two possible explanations for the decline in alpha diversity measures. The first is derived from “area heterogeneity trade-off hypothesis” [36]. We found significantly higher habitat heterogeneity during the third vegetation census than in the past. According to the aforementioned hypothesis, this increase in habitat heterogeneity could decrease the resource availability and increase the local extinction of the species, especially those associated with typical beech forest habitats. Furthermore, it could have been caused by the reduction of the total abundance of some species across the communities and, consequently, their rarer occurrence under the influence of high-intensity forest use [51].

**Figure 6:**
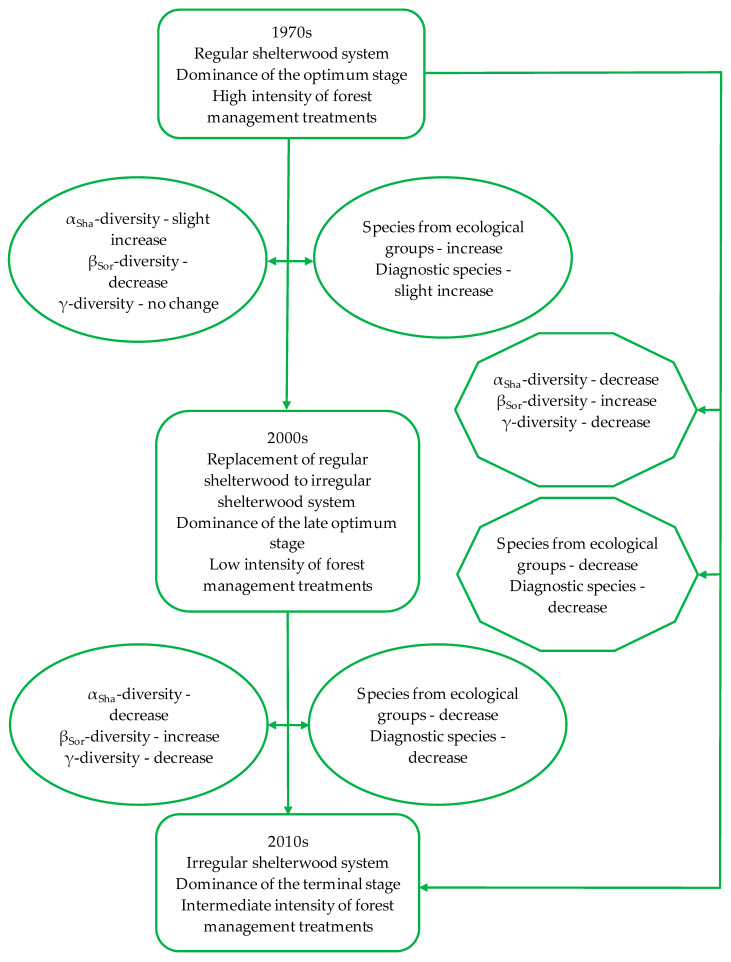
Impact of the forest management and stand structure on the herbaceous plant diversity in Carpathian beech forests with over 40 years of forest development—comparison of the results obtained on the basis of records from three and two time points.

Since the findings on long-term changes in the diversity of herbaceous forest plants are most often based on a comparison of vegetation records from two censuses, they can only show an increase, a decrease, or no change between records. To reliably recognize the patterns of plant diversity dynamics, a time series of vegetation data recorded on permanent sampling plots are needed [23]. Our research showed that over 40 years, alpha diversity decreased, and the total beta diversity increased. However, this pattern of changes was consistent with the course of changes in the second study period (between the 2000s and 2010s). At the same time, it was significantly different from the pattern of changes recorded in the first, three times longer study period (30 years between the 1970s and 2000s). Thus, our results indicated that the temporal changes in diversity detected by comparing the records from the two censuses may (1) hide the actual course of the changes and/or (2) contribute to the misinterpretation of the trend of long-term changes in the herbaceous plant diversity and, consequently, lead to the incorrect assessment of the state of forest diversity or conservation action plans (Figure 6). This is well-evidenced by the contrast between an increase in beta diversity and the decline in the share of diagnostic species of herbaceous vegetation of FMBF. The occurrence of a diagnostic species depends on the degree of conservation of habitat conditions, typical for a well-preserved community. Hence, the decline of diagnostic species for beech forests indicates the decay of habitats typical of well-preserved FMBF. It is an open question that requires further research, whether the disappearance of these species is related exclusively to the final developmental stage of the managed forest (terminal stage) or whether it is of a permanent, irreversible nature (independent of developmental stage). Thus, our results confirm the great importance of conservation-relevant species as an important metric of the conservation status of forests [11].

### 4.2. Impact of Changes in Forest Structure as Well Intensity of Forest Management on Herbaceous Plant Diversity Metrics

During the first study period, the age of the forest increased, and the regular shelterwood was replaced by closer-to-nature, irregular shelterwood. As a result of the aging of the forest, the species richness of the stand decreased, and the DBH (and DBH variability) increased. Additionally, the changes of the management system resulted in a decrease in intensity of the forest management treatment. At the same time, diversity metrics of herbaceous plants slightly increased within-plots and significantly decreased between-plots. In the second study period, along with the growing age of the stands, there were significant changes in the structure of the tree and shrub layers. Moreover, because of the change of the dominant stage of the stand development from the optimum to terminal, the intensity of the forest management treatments increased. At the same time, the diversity metrics of the herbaceous layer significantly decreased within-plots and increased between-plots.

We argue, similarly to Dieler et al. [9] and Hilmers et al. [10], that the revealed pattern of diversity was a response to complex changes in the forest management affecting the forest structure and development status. The results obtained by Schall et al. [13] suggest a negative impact of the closer-to-nature management system on the spatial diversity of the forest habitats and, thus, on beta diversity. Indeed, after replacing the regular with an irregular shelterwood system (first study period), we found a decline in beta diversity (between-plot diversity). However, we did not find significant changes in the habitat heterogeneity, nor with the alpha diversity (within-plot diversity). Moreover, we did not find a lower beta diversity in forests managed by the closer-to-nature management system during the second study period; in fact, it was higher (except beta nestedness). At the end of the second study period, the habitat heterogeneity was also greater, however, this did not result in an increase in the alpha diversity, which decreased significantly due to the high density of tree regeneration (in this work, represented by the shrub layer). This means that the introduction of a closer-to-nature management system did not result in decreased heterogeneity of the habitats and herbaceous plant beta diversity. We argue that this was the result of intensified renewal processes taking place in the terminal stage of stands development, which increased the forest habitat heterogeneity. At the same time, the intensive forest management related to tree removal and the development of new tree generation caused the decline in alpha diversity metrics. This decline in alpha diversity enhanced the effect of increased beta diversity. Thus, recorded during a 40-year time period, the increase in beta diversity can be strongly misleading from the point of view of the forest conservation status assessment.

## 5. Conclusions

Our results showed that the herbaceous plant diversity is highly dependent not only on the forest management system but, also, on the intensity of forest treatments [13]. We showed the importance of temporal interactions between the changes in the management system—the dominant development stage—the intensity of the forest treatments, and the herbaceous plant diversity. Hence, it should be assumed that different levels of intensity of these interactions will result in different patterns of change in herbaceous plant diversity. In view of the complexity of the processes affecting the herbaceous plant diversity in FMBF, it would be a mistake to reject or prioritize alpha or beta diversity measurements to determine the diversity dynamics or assess the conservation status of these forests. We argue that, in order to reveal the real course of long-term changes in herbaceous plant diversity and to properly assess the state of forest biodiversity as well as conservation status or action plans, we need to learn more about the long-term interactions between drivers that control the forest structure and the various measures of plant diversity.

## Figures and Tables

**Table 1:** Characteristics of the forest management methods used in the Sanocko-Turczańskie Mountains beech forests.

	Regular Shelterwood System	Irregular Shelterwood Systems
Rotation age	80–110 years	110–130 years
Regeneration period	10–20 years	30–50 years
Regeneration processes	System in which, in order to provide a source of seed and/or protection for regeneration, the mature stand is removed in two or more overstory removal cuttings. The first of which is an establishment cutting to establish the regeneration from the seeds. After 2–5 years, to provide the best conditions for the growth of a new generation of trees partial mature trees, removals are started. After 10–20 years, all the mature trees are removed by a final cut.	In dense stands, foresters choose irregularly distributed plots where, every 3–6 years, they cut a small group of trees, forming small gaps. This cycle is repeated within the previously formed gaps, where another small group of trees are cut, thus expanding the gaps in the stands. Process of expanding the gaps continues throughout the regeneration cycle.
Stand structure	even-aged	uneven-aged

**Table 2:** Mean (±SE) values of forest structure characteristics in three subsequent vegetation censuses. Differences between vegetation censuses were tested by several sample repeated measures tests. Depending on normality distribution, an ANOVA or Friedman test was used. Values with different superscript letters differed significantly based on Tukey’s or Wilcoxon’s posteriori tests at the *p* level, at least *p* ≤ 0.05. Intensity of forest management treatments were ranked on a five-point scale as follows: no interference—1, thinning—4, irregular shelterwood treatments lasting 10 years—6, irregular shelterwood treatments lasting 20 years—7, and regular shelterwood treatments—9 (for details, please refer to the Materials and Methods section). F and Chi^2^—ANOVA and Friedman test score, respectively.

	Test Score	Mean (±SE) Values		
	F, ^x^ Chi^2^	1970s	2000s	2010s
Cover of tree layer (%)	^x^6.7 *	87.3 (1.02) ^a^	84.0 (1.16) ^a^	77.6 (2.60) ^b^
Cover of shrub layer (%)	^x^40.0 ***	5.6 (0.63) ^a^	8.8 (1.45) ^a^	27.2 (2.77) ^b^
Average tree height (m)	^x^11.1 **	30.3 (0.47) ^a^	31.0 (0.55) ^a^	27.1 (0.73) ^b^
Average DBH (cm)	6.3 **	37.9 (1.43) ^a^	49.0 (3.51) ^b^	40.1 (2.35) ^a^
Tree layer species richness (No. of species)	^x^27.5 ***	2.9 (0.15) ^a^	2.3 (0.11) ^b^	1.8 (0.08) ^c^
Shrub layer species richness (No. of species)	3.5 *	1.7 (0.11) ^a^	2.3 (0.17) ^ab^	2.3 (0.15) ^b^
Age of stands (year)	74.0 ***	85.3 (2.45) ^a^	96.3 (2.50) ^b^	113.0 (2.49) ^c^
Intensity of forest management (ranks)	5.3 **	6.5 (0.31) ^a^	5.4 (0.28) ^b^	5.7 (0.25) ^ab^

* *p* ≤ 0.05. ** *p* ≤ 0.01. *** *p* ≤ 0.001.

**Table 3:** Mean (±SE) values of herbaceous layer characteristics in three subsequent vegetation censuses. Differences between vegetation censuses were tested by several sample repeated measures tests. Depending on the normality distribution, an ANOVA or Friedman test was used. Values with different superscript letters (a,b,c) differed significantly based on Tukey’s or Wilcoxon’s posteriori tests at the *p* level, at least *p* ≤ 0.05. F and Chi^2^—ANOVA and Friedman test scores, respectively. L, T, F, R, and N: Ellenberg indicator values for light, temperature, moisture, reaction, and nitrogen. The L and H subscripts indicate low and high indicator values, respectively.

	Test Score	Mean (±SE) Values		
	F, ^x^Chi^2^	1970s	2000s	2010s
Frequency of species occurrence	^x^23.2 ***	16.0 (±1.8) ^a^	17.4 (±1.9) ^a^	11.5 (±1.5) ^b^
Species richness (No. of species)	42.5 ***	27.9 (±1.0) ^a^	30.2 (±1.0) ^a^	20.1 (±0.7) ^b^
Total abundance of species (%)	^x^26.5 ***	86.2 (±4.5) ^a^	98.1 (±3.8) ^a^	128.7 (±6.8) ^b^
Number of species with high or low habitat requirements				
L_L_	56.2 ***	8.9 (±0.3) ^a^	10.1 (±0.3) ^b^	6.2 (±0.3) ^c^
L_H_	2.7	2.4 (±0.2) ^a^	2.8 (±0.2) ^a^	2.7 (±0.2) ^a^
T_L_	^x^27.1 ***	2.1 (±0.2) ^a^	2.4 (±0.2) ^a^	1.4 (±0.1) ^b^
T_H_	2.3	1.6 (±0.2) ^a^	1.9 (±0.2) ^a^	1.6 (±0.2) ^a^
F_H_	^x^32.6 ***	2.7 (±0.2) ^a^	3.8 (±0.2) ^b^	1.8 (±0.2) ^c^
R_L_	^x^1.8	0.2 (±0.1) ^a^	0.4 (±0.1) ^a^	0.3 (±0.1) ^a^
R_H_	25.2 ***	10.4 (± 0.5) ^a^	10.5 (±0.5) ^a^	7.0 (±0.4) ^b^
N_L_	0.6	0.4 (±0.1) ^a^	0.4 (±0.1) ^a^	0.3 (±0.1) ^a^
N_H_	19.5 ***	9.7 (±0.5) ^a^	10.7 (±0.6) ^a^	7.0 (±0.4) ^b^

*** *p* ≤ 0.001.

**Table 4:** Correlation between changes in the forest structure characteristics and changes in the herbaceous plant diversity metrics, expressed by Spearman’s correlation coefficients. The correlation coefficients at the *p* level, at least *p* ≤ 0.05, have been highlighted in grey and italicized. In the case of groups of species with high and low habitat requirements, the groups that recorded significant differences in the species richness between the vegetation censuses (according to the results provided in Table 3) were selected for correlation.

	∆ Cover of Tree Layer (%)	∆ Cover of Shrub Layer (%)	∆ Average Tree Height (m)	∆ Average DBH (cm)	∆ Tree Layer Species Richness (No. of Species)	∆ Shrub Layer Species Richness (No. of Species)	∆ Age of Stands (Year)
First study period							
∆α_Sha_	0.08	−0.21	0.06	−0.03	0.19	0.04	0.05
∆Evenness	0.12	−0.01	−0.05	−0.2	0.1	−0.15	−0.05
∆L_L_	0.21	−0.17	0.05	−0.18	0.1	0.03	−0.09
∆T_L_	−0.07	−0.19	0.04	0.07	0.02	−0.04	0.07
∆F_H_	−0.13	−0.17	0.06	0.12	0.18	0.02	0.17
∆R_H_	−0.03	−0.16	0.08	0.04	0.04	0.09	0,00
∆N_H_	−0.06	−0.15	0.01	0,00	0.09	0.12	−0.04
∆Species richness	0.02	−0.19	0.05	−0.02	0.15	0.11	0.01
∆β_Sor_	0,00	0.15	−0.18	−0.06	−0.14	−0.08	−0.06
∆β_Sim_	−0.02	0.07	0,00	0,00	−0.02	0.04	0.04
∆β_nes_	0.04	0.03	−0.14	0.06	−0.08	−0.07	−0.09
Second study period							
∆α_Sha_	−0.05	*−0.26*	0.18	−0.06	0.03	0.05	−0.07
∆Evenness	−0.03	−0.07	−0.01	−0.17	−0.12	*−0.26*	−0.2
∆L_L_	0.2	*−0.3*	−0.08	−0.23	0.07	−0.09	0,00
∆T_L_	−0.12	−0.05	0.19	0.03	0.03	0.06	−0.07
∆F_H_	−0.09	*−0.3*	0.08	−0.08	0.14	0.13	−0.18
∆R_H_	0.04	*−0.35*	0.09	−0.06	0.18	0.12	−0.03
∆N_H_	0.07	*−0.4*	0.07	−0.1	0.15	0.14	−0.04
∆Species richness	0.02	*−0.37*	0.18	−0.11	0.17	0.08	−0.07
∆β_Sor_	−0.08	0.04	*−0.25*	0.02	*−0.26*	−0.13	0.05
∆β_Sim_	*−0.3*	0.01	−0.08	0.21	−0.24	0.12	0.11
∆β_nes_	*0.27*	0.04	0.02	−0.1	0.05	*−0.24*	−0.07

## Data Availability

The data presented in this study are available in the article. Additional data are available on request from the corresponding author.

## Supplementary Materials

The following are available online at https://www.mdpi.com/article/10.3390/biology10050406/s1, Table S1: Herbaceous species with more than 10% changes in frequency of occurrence and/or significant changes in abundance. Increases in frequency of occurrence and/or abundance of species are marked in bold and decreases by underlining. L, T, F, R, N: Ellenberg’s indicator values for light, temperature, moisture, reaction and nitrogen.

## References

[1] Gilliam F.S. (2007). The ecological significance of the herbaceous layer in temperate forest ecosystems. BioScience.

[2] Rackham O. (2008). Ancient woodlands: Modern threats. New Phytol..

[3] Bengtsson J., Nilsson S.G., Franc A., Menozzi P. (2000). Biodiversity, disturbances, ecosystem function and management of European forests. For. Ecol. Manag..

[4] Perring M.P., Bernhardt-Römermann M., Baeten L., Midolo G., Blondeel H., Depauw L., Landuyt D., Maes S.L., De Lombaerde E., Carón M.M. (2018). Global environmental change effects on plant community composition trajectories depend upon management legacies. Glob. Chang. Biol..

[5] Bürgi M., Li L., Kizos T. (2015). Exploring links between culture and biodiversity: Studying land use intensity from the plot to the landscape level. Biodivers. Conserv..

[6] Franklin J., Serra-Diaz J., Syphard A., Regan H. (2016). Global change and terrestrial plant community dynamics. Proc. Nat. Acad. Sci. USA.

[7] Naaf T., Wulf M. (2010). Habitat specialists and generalists drive homogenization and differentiation of temperate forest plant communities at the regional scale. Biodivers. Conserv..

[8] Kopecký M., Hédl R., Szabó P. (2013). Non-random extinctions dominate plant community changes in abandoned coppices. J. Appl. Ecol..

[9] Dieler J., Uhl E., Biber P., Müller J., Rötzer T., Pretzsch H. (2017). Effect of forest stand management on species composition, structural diversity, and productivity in the temperate zone of Europe. J. For. Res..

[10] Hilmers T., Friess N., Bässler C., Heurich M., Brandl R., Pretzsch H., Seidl R., Müller J. (2018). Biodiversity along temperate forest succession. J. Appl. Ecol..

[11] Lelli C., Bruun H.H., Chiaruccia A., Donatia D., Frascarolia F., Fritz O., Goldberg I., Nascimbene J., Tøttrup A., Rahbek C. (2019). Biodiversity response to forest structure and management: Comparing species richness, conservation relevant species and functional diversity as metrics in forest conservation. For. Ecol. Manag..

[12] Durak T., Bugno-Pogoda A., Durak R. (2021). Application of forest inventories to assess the forest developmental stages on plots dedicated to long-term vegetation studies. For. Ecol. Manag..

[13] Schall P., Gossner M.M., Heinrichs S., Fischer M., Boch S., Prati D., Jung K., Baumgartner V., Blaser S., Böhm S. (2018). The impact of even-aged and uneven-aged forest management on regional biodiversity of multiple taxa in European beech forests. J. Appl. Ecol..

[14] Decocq G., Aubert M., Dupont F., Alard D., Saguez R., Wattez-Franger A., de Foucault B., Delelis-Dusollier A., Bardat J. (2004). Plant diversity in a managed temperate deciduous forest: Understorey response to two silvicultural systems. J. Appl. Ecol..

[15] Ciancio O., Corona P., Lamonaca A., Portoghesi L., Travaglini D. (2006). Conversion of clearcut beech coppices into high forests with continuous cover: A case study in central Italy. For. Ecol. Manag..

[16] Puettmann K.J., Wilson S.M., Baker S.C., Donoso P.J., Drössler L., Amente G., Harvey B.D., Knoke T., Lu Y., Nocentini S. (2015). Silvicultural alternatives to conventional even-aged forest management—what limits global adoption?. For. Ecosyst..

[17] O’Hara K.L. (2016). What is close-to-nature silviculture in a changing world?. Forestry.

[18] Decocq G., Aubert M., Dupont F., Bardat J., Wattez-Franger A., Saguez R., de Foucault B., Alard D., Delelis-Dusollier A. (2005). Silviculture-driven vegetation change in a European temperate deciduous forest. Ann. For. Sci..

[19] Baeten L., Bauwens B., De Schrijver A., De Keersmaeker L., Van Calster H., Vandekerkhove K., Roelandt B., Beeckman H., Verheyen K. (2009). Herb layer changes (1954–2000) related to the conversion of coppice-withstandards forest and soil acidification. Appl. Veg. Sci..

[20] Müllerová J., Hédl R., Szabó P. (2015). Coppice abandonment and its implications for species diversity in forest vegetation. For. Ecol. Manag..

[21] Durak T., Holeksa J. (2015). Biotic homogenisation and differentiation along a habitat gradient resulting from the ageing of manager beech stands. For. Ecol. Manag..

[22] FOREST EUROPE 2020: State of Europe’s Forests 2020. https://foresteurope.org/wp-content/uploads/2016/08/SoEF_2020.pdf.

[23] Bakker J.P., Olff H., Willems J.H., Zobel M. (1996). Why do we need permanent plots in the study of long-term vegetation dynamics?. J. Veg. Sci..

[24] De Lombaerde E., Verheyen K., Perring M.P., Bernhardt-Römermann M., Van Calster H., Brunet J., Chudomelová M., Decocq G., Diekmann M., Durak T. (2018). Responses of competitive understorey species to spatial environmental gradients inaccurately explain temporal changes. Basic Appl. Ecol..

[25] Van Calster H., Baeten L., De Schrijver A., De Keersmaeker L., Rogister J.E., Verheyen K., Hermy M. (2007). Management driven changes (1967–2005) in soil acidity and the understorey plant community following conversion of a coppice-with-standards forest. For. Ecol. Manag..

[26] Durak T., Durak R. (2015). Vegetation changes in meso-and eutrophic submontane oak–hornbeam forests under long-term high forest management. For. Ecol. Manag..

[27] Heinrichs S., Schmidt W. (2017). Biotic homogenization of herb layer composition between two contrasting beech forest communities on limestone over 50 years. Appl. Veg. Sci..

[28] Paillet Y., Bergès L., Hjaltén J., Ódor P., Avon C., Bernhardt-Römermann M., Bijlsma R.J., De Bruyn L., Fuhr M., Grandin U. (2010). Biodiversity differences between managed and unmanaged forests: Meta analysis of species richness in Europe. Conserv. Biol..

[29] Chaudhary A., Burivalova Z., Koh L.P., Hellweg S. (2016). Impact of forest management on species richness: Global meta-analysis and economic trade-offs. Sci. Rep..

[30] Gotelli N.J., Colwell R.K. (2001). Quantifying biodiversity: Procedures and pitfalls in the measurement and comparison of species richness. Ecol. Lett..

[31] McKinney M.L., Lockwood J.L. (1999). Biotic homogenization: A few winners replacing many losers in the next mass extinction. Trends Ecol. Evol..

[32] Olden J.D., Rooney T.P. (2006). On defining and quantifying biotic homogenization. Glob. Ecol. Biogeogr..

[33] Whittaker R.H. (1970). Evolution and measurement of species diversity. Taxon.

[34] Loreau M. (2000). Are communities saturated? On the relationship between α, β, and ϒ diversity. Ecol. Lett..

[35] Melo A.S., Fernando T., Rancel L.V.B., Diniz-Filho J.A.F. (2009). Environmental drivers of beta-diversity patterns in New-World birds and mammals. Ecography.

[36] Allouche O., Kalyuzhny M., Moreno-Rueda G., Pizarro M., Kadmon R. (2012). Area-heterogeneity tradeoff and the diversity of ecological communities. Proc. Nat. Acad. Sci. USA.

[37] Dzwonko Z. (1977). Forest communities of the Gory Słonne Range (Polish Eastern Carpathians). Fragm. Flor. Geobot..

[38] Skiba S., Drewnik M. (2003). Soil map of the polish carpathian mountains. Rocz. Bieszcz..

[39] Meteorological Data Averages and Monthly Totals. https://meteomodel.pl/dane/srednie-miesieczne/?imgwid=349220690&par=tm&max_empty=2.

[40] Jaworski A., Kołodziej Z. (2004). Beech (Fagus sylvatica L.) forest of a selection structure in the Bieszczady Mountains (southeastern Poland). J. For. Sci..

[41] Braun-Blanquet J. (1964). Pflanzensoziologie. Grundzüge der Vegetationskunde.

[42] Baselga A. (2010). Partitioning the turnover and nestedness components of beta diversity. Glob. Ecol. Biogeogr..

[43] Rooney T.P., Wiegmann S.M., Rogers D.A., Waller D.M. (2004). Biotic impoverishment and homogenization in unfragmented forest understory communities. Conserv. Biol..

[44] Durak T., Durak R., Węgrzyn E., Leniowski K. (2015). The impact of changes in species richness and species replacement on patterns of taxonomic homogenization in the Carpathian forest ecosystems. Forests.

[45] Olden J.D., Poff N.L. (2003). Toward a mechanistic understanding and prediction of biotic homogenization. Am. Nat..

[46] Ellenberg H., Weber H.E., Düll R., Wirth V., Werner W., Paulissen D. (1992). Zeigerwerte von pflanzen in Mitteleuropa. Scr. Geobot..

[47] Horn H.S. (1966). Measurement of overlap in comparative ecological studies. Am. Nat..

[48] Matuszkiewicz W. (2001). Przewodnik do Oznaczania Zbiorowisk Roślinnych Polski. [A Guide for the Identification of Polish Plant Communities].

[49] Hammer O., Harper D.A.T., Ryan P.D. (2001). PAST: Paleontological statistics software package for education and data analysis. Palaeontol. Electron..

[50] Jost L. (2007). Partitioning diversity into independent alpha and beta components. Ecology.

[51] Karp D.S., Rominger A.J., Zook J., Ranganathan J., Ehrlich P.R., Daily G.C. (2012). Intensive agriculture erodes b-diversity at large scales. Ecol. Lett..

