# Supplementary material for: Impact of Forest Management on the Temporal Dynamics of Herbaceous Plant Diversity in the Carpathian Beech Forests over 40 Years
Source: Biology (Basel). 2021 May 5;10(5):406. doi: 10.3390/biology10050406 (PMC8147971; doi:10.3390/biology10050406)
Supplement: Supplementary file 1 [file biology-10-00406-s001.zip › biology-1175270-supplementary.pdf]

Table S1. Herbaceous species with more than 10% changes in frequency of occurrence and/or significant changes in abundance. Increases in frequency of occurrence and/or abundance of species are marked in bold and decreases by underlining. L, T, F, R, N: Ellenberg's indicator values for light, temperature, moisture, reaction and nitrogen.

	Frequency of occurences (%)			Changes in frequency of occurence (%)			Total abundance (sum of the mid-point percentage cover values)			Changes in abundance (%)			Ellenberg indicator values (EIVs)				
	1970s	2000s	2010s	First study period	Second study period	Total study period	1970s	2000s	2010s	First study period	Second study period	Total study period	L	T	F	R	N
Winners																	
Acer platanoides	1.5	41.8	10.4	40.3	-31.3	**9.0**	0.5	16.5	16.0	16.0	-0.5	**15.5**	4	6	x	x	x
Anemone nemorosa	52.2	88.1	59.7	35.8	-28.4	**7.5**	212.5	519.0	336.0	336.5	-183.0	**123.5**	x	x	5	x	x
Carex sylvatica	26.9	50.7	34.3	23.9	-16.4	**7.5**	9.0	24.5	66.0	15.5	41.5	**57.0**	2	5	5	6	5
Dryopteris sp.	86.6	94.0	95.5	7.5	1.5	**9.0**	44.0	73.5	434.0	29.5	361.0	**390.5**	-	-	-	-	-
Impatiens parviflora	0.0	13.4	7.5	13.4	-6.0	**7.5**	0.0	9.5	24.5	9.5	15.0	**24.5**	4	6	5	x	6
Juncus effusus	0.0	1.5	10.4	1.5	9.0	**10.4**	0.0	0.5	25.5	0.5	25.0	**25.5**	8	5	7	3	4
Lysimachia nemorum	0.0	26.9	10.4	26.9	-16.4	**10.4**	0.0	11.5	25.5	11.5	14.0	**25.5**	2	5	7	7	7
Lysimachia vulgaris	0.0	0.0	9.0	0.0	9.0	**9.0**	0.0	0.0	25.0	0.0	25.0	**25.0**	6	x	8	x	x
Platanthera bifolia	0.0	0.0	7.5	0.0	7.5	**7.5**	0.0	0.0	5.0	0.0	5.0	**5.0**	6	x	5	7	x
Rubus hirtus	67.2	79.1	85.1	11.9	6.0	**17.9**	52.5	552.5	569.0	500.0	16.5	**516.5**	7	4	5	x	x
Rumex obtusifolius	0.0	4.5	14.9	4.5	10.4	**14.9**	0.0	1.5	17.5	1.5	16.0	**17.5**	7	5	6	x	9
Salvia glutinosa	56.7	53.7	65.7	-3.0	11.9	**9.0**	24.0	35.0	216.0	11.0	181.0	**192.0**	4	5	6	7	7
Anthriscus sylvestris	0.0	0.0	10.4	0.0	10.4	**10.4**	0.0	0.0	25.5	0.0	25.5	25.5	7	x	5	x	8
Acer pseudoplatanus	71.6	88.1	73.1	16.4	-14.9	1.5	29.0	110.5	230.0	81.5	119.5	**201.0**	4	x	6	x	7
Galium odoratum	79.1	76.1	74.6	-3.0	-1.5	-4.5	239.0	261.0	573.0	22.0	312.0	**334.0**	2	5	5	6	5
Hieracium murorum	0.0	0.0	6.0	0.0	6.0	6.0	0.0	0.0	7.0	0.0	7.0	**7.0**	-	-	-	-	-
Impatiens noli-tangere	58.2	64.2	59.7	6.0	-4.5	1.5	310.0	203.0	584.0	-107.0	381.0	**274.0**	4	5	7	7	6
Polystichum braunii	0.0	1.5	6.0	1.5	4.5	6.0	0.0	0.5	9.5	0.5	9.0	**9.5**	3	4	6	6	7
Senecio sp.	65.7	74.6	67.2	9.0	-7.5	1.5	24.5	32.5	146.0	8.0	113.5	**121.5**	7	-	-	x	8
Stellaria holostea	7.5	13.4	13.4	6.0	0.0	6.0	2.5	21.5	60.5	19.0	39.0	**58.0**	5	6	5	6	5
Veronica montana	23.9	58.2	17.9	34.3	-40.3	-6.0	13.0	19.5	33.5	6.5	14.0	**20.5**	4	5	7	5	6
Losers																	
Anthriscus nitida	22.4	31.3	1.5	9.0	-29.9	-20.9	7.5	27.5	3.0	20.0	-24.5	-4.5	4	4	6	8	8
Arum alpinum	10.4	1.5	1.5	-9.0	0.0	-9.0	3.5	0.5	0.5	-3.0	0.0	-3.0	-	-	-	-	-
Athyrium filix-femina	89.6	98.5	23.9	9.0	-74.6	-65.7	128.5	330.0	67.0	201.5	-263.0	-61.5	3	x	7	x	6
Chaerophyllum aromaticum	10.4	3.0	0.0	-7.5	-3.0	-10.4	3.5	1.0	0.0	-2.5	-1.0	-3.5	7	5	7	6	8
Chrysosplenium alternifolium	17.9	1.5	0.0	-16.4	-1.5	-17.9	6.0	0.5	0.0	-5.5	-0.5	-6.0	4	4	8	7	5
Corylus avellana	35.8	19.4	11.9	-16.4	-7.5	-23.9	12.0	6.5	9.0	-5.5	2.5	-3.0	6	5	x	x	5
Daphne mezereum	44.8	10.4	3.0	-34.3	-7.5	-41.8	15.0	3.5	1.0	-11.5	-2.5	-14.0	4	x	5	7	5
Galeopsis speciosa	7.5	22.4	0.0	14.9	-22.4	-7.5	2.5	7.5	0	5.0	-7.5	-2.5	7	x	5	x	8
Gentiana asclepiadea	11.9	1.5	4.5	-10.4	3.0	-7.5	6.5	0.5	4.0	-6.0	3.5	-2.5	7	x	6	7	2
Geranium phaeum	23.9	11.9	3.0	-11.9	-9.0	-20.9	8.0	4.0	6.0	-4.0	2.0	-2.0	6	x	5	6	5
Glechoma hirsuta	52.2	55.2	20.9	3.0	-34.3	-31.3	52.5	31.0	51.5	-21.5	20.5	-1.0	-	-	-	-	-
Lonicera xylosteum	23.9	6.0	0.0	-17.9	-6.0	-23.9	8.0	2.0	0.0	-6.0	-2.0	-8.0	5	6	5	7	6
Mercurialis perennis	74.6	71.6	53.7	-3.0	-17.9	-20.9	409.5	175.5	307.0	-234.0	131.5	-102.5	2	x	x	8	7
Oxalis acetosella	65.7	74.6	41.8	9.0	-32.8	-23.9	314.5	71.5	109.5	-243.0	38.0	-205.5	1	x	5	4	6
Phyteuma spicatum	10.4	4.5	0.0	-6.0	-4.5	-10.4	3.5	1.5	0.0	-2.0	-1.5	-3.5	x	x	5	6	5
Polygonatum multiflorum	55.2	44.8	13.4	-10.4	-31.3	-41.8	18.5	15.0	9.5	-3.5	-5.5	-9.0	2	x	5	6	5
Populus tremula	11.9	1.5	1.5	-10.4	0.0	-10.4	4.0	0.5	0.5	-3.5	0.0	-3.5	6	5	5	x	x
Primula elatior	9.0	6.0	0.0	-3.0	-6.0	-9.0	3.0	2.0	0.0	-1.0	-2.0	-3.0	6	x	6	7	7
Ribes uva-crispa	40.3	25.4	7.5	-14.9	-17.9	-32.8	13.5	8.5	10.0	-5.0	1.5	-3.5	4	5	x	x	6
Sambucus racemosa	11.9	14.9	3.0	3.0	-11.9	-9.0	4.0	5.0	3.5	1.0	-1.5	-0.5	6	4	5	5	8
Sanicula europaea	28.4	10.4	7.5	-17.9	-3.0	-20.9	9.5	6.0	7.5	-3.5	1.5	-2.0	4	5	5	8	6
Stellaria nemorum	19.4	40.3	0.0	20.9	-40.3	-19.4	9.0	13.5	0.0	4.5	-13.5	-9.0	4	x	7	5	7
Symphytum tuberosum	11.9	10.4	1.5	-1.5	-9.0	-10.4	4.0	20.5	3.0	16.5	-17.5	-1.0	5	8	5	6	7
Viburnum opulus	13.4	0.0	0.0	-13.4	0.0	-13.4	4.5	0.0	0.0	-4.5	0.0	-4.5	6	5	x	7	6

Table 4. Continuation.

	Frequency of occurences (%)			Changes in frequency of occurence (%)			Total abundance (sum of the mid-point percentage cover values)			Changes in abundance (%)			Ellenberg indicator values (EIVs)				
	1970s	2000s	2010s	First study period	Second study period	Total study period	1970s	2000s	2010s	First study period	Second study period	Total study period	L	T	F	R	N
Viola reichenbachiana	19.4	23.9	9.0	4.5	-14.9	-10.4	9.0	8.0	5.5	-1.0	-2.5	-3.5	4	x	5	7	6
Abies alba	71.6	70.1	50.7	-1.5	-19.4	-20.9	26.5	23.5	141	-3.0	117.0	114.0	3	5	x	x	x
Actaea spicata	61.2	41.8	17.9	-19.4	-23.9	-43.3	20.5	14	38	-6.5	24.0	17.5	3	5	5	6	7
Carex pilosa	53.7	47.8	40.3	-6.0	-7.5	-13.4	687.5	847.5	730.5	160.0	-117.0	43.0	4	6	5	5	5
Circaea lutetiana	46.3	56.7	38.8	10.4	-17.9	-7.5	18.0	33.5	113.5	15.5	80.0	95.5	4	5	6	7	7
Dentaria bulbifera	91.0	91.0	74.6	0.0	-16.4	-16.4	320	463	323	142.5	-140.0	2.5	3	5	5	7	6
Euphorbia amygdaloides	26.9	26.9	14.9	0.0	-11.9	-11.9	11.5	11.5	20.0	0.0	8.5	8.5	4	5	5	8	5
Fraxinus excelsior	38.8	61.2	10.4	22.4	-50.7	-28.4	15.5	93.5	28.0	78.0	-65.5	12.5	4	5	x	7	7
Milium effusum	46.3	47.8	35.8	1.5	-11.9	-10.4	15.5	21.0	54.0	5.5	33.0	38.5	4	x	5	5	5
Paris quadrifolia	44.8	56.7	25.4	11.9	-31.3	-19.4	15.0	19.0	26.0	4.0	7.0	11.0	3	x	6	7	7
Petasites albus	46.3	28.4	20.9	-17.9	-7.5	-25.4	106.5	43.5	158.0	-63.0	114.5	51.5	4	4	6	x	5
Prenanthes purpurea	9.0	9.0	1.5	0.0	-7.5	-7.5	3.0	3.0	3.0	0.0	0.0	0.0	4	4	5	5	5
Scrophularia nodosa	16.4	14.9	4.5	-1.5	-10.4	-11.9	5.5	5.0	6.5	-0.5	1.5	1.0	4	5	6	6	7
Symphytum cordatum	68.7	68.7	59.7	0.0	-9.0	-9.0	243.5	281.0	160.5	37.5	-120.5	-83.0	-	-	-	-	-
Ulmus glabra	23.9	35.8	10.4	11.9	-25.4	-13.4	22.5	12.0	13.5	-10.5	1.5	-9.0	4	5	6	7	7
Carex digitata	4.5	9.0	0.0	4.5	-9.0	-4.5	1.5	3.0	0.0	1.5	-3.0	-1.5	3	x	5	x	4
Cerasus avium	1.5	6.0	0.0	4.5	-6.0	-1.5	0.5	2.0	0.0	1.5	-2.0	-0.5	-	-	-	-	-
Circaea alpina	4.5	7.5	0.0	3.0	-7.5	-4.5	1.5	2.5	0.0	1.0	-2.5	-1.5	4	4	7	5	5
Dentaria glandulosa	61.2	46.3	61.2	-14.9	14.9	0.0	275.0	86.5	220.5	-188.5	134.0	-54.5	-	-	-	-	-
Euonymus europaea	6.0	13.4	0.0	7.5	-13.4	-6.0	2.0	4.5	0.0	2.5	-4.5	-2.0	6	5	5	8	5
Rubus idaeus	3.0	14.9	0.0	11.9	-14.9	-3.0	1.0	19.5	0.0	18.5	-19.5	-1.0	7	x	x	x	6
Mixed reaction																	
Fagus sylvatica	59.7	92.5	38.8	32.8	-53.7	-20.9	39.5	53.0	120.5	13.5	67.5	**81.0**	3	5	5	x	x
Galeobdolon luteum	92.5	95.5	83.6	3.0	-11.9	-9.0	258.5	480.0	714.5	221.5	234.5	**456.0**	-	-	-	-	-
Geranium robertianum	52.2	19.4	20.9	-32.8	1.5	-31.3	20.0	6.5	53.5	-13.5	47.0	**33.5**	5	x	x	x	7
Pulmonaria obscura	73.1	58.2	14.9	-14.9	-43.3	-58.2	49.5	19.5	54.0	-30.0	34.5	**4.5**	4	5	6	8	7
